# Physical Activity in the Southern Great Plain Region of Hungary: The Role of Sociodemographics and Body Mass Index

**DOI:** 10.3390/ijerph182312414

**Published:** 2021-11-25

**Authors:** Ferenc Győri, Tamás Berki, Zoltán Katona, Beáta Vári, Zsolt Katona, Zita Petrovszki

**Affiliations:** Institution of Physical Education and Sport Sciences, Faculty of Education, University of Szeged, 6720 Szeged, Hungary; gyori.ferenc.jozsef@szte.hu (F.G.); katona.zoltan@szte.hu (Z.K.); vari.beata@szte.hu (B.V.); katona.zsolt.zoltan@szte.hu (Z.K.); hajdune.petrovszki.zita@szte.hu (Z.P.)

**Keywords:** IPAQ, Southern Great Plain, Hungary, physical activity, sociodemographic

## Abstract

This study explores the level of physical activity and its associations with sociodemographics and body mass index (BMI) in the Southern Great Plain region of Hungary. A total of 1648 adults (Men = 572; Women = 1076) were involved in this study. Their mean age was 43.0 (SD = 15.3), and they were recruited at different face-to-face events from July 2018 to January 2019. The International Physical Activity Questionnaire (IPAQ) was used to assess physical activity, and the participants were asked different questions related to their sociodemographics (e.g., education, income) and physical attributes (e.g., height, weight). Additionally, a descriptive statistical, chi-square test was used the see the gender differences, and multinominal regression analysis was used to see the associations between gender, age, place of residence, education, income, BMI, and physical activity levels. Our analysis showed that 19.2% of the sample had a low-, 41.1% had a moderate-, and 39.7% had a high level of physical activity. Furthermore, a high and a moderate level of physical activity were associated with gender, age, residence, education, and BMI. We believe this present study helps understand the role of physical activity in health through the example of the Southern Great Plain region of Hungary, which can provide useful information for experts to increase participation in regular physical activity.

## 1. Introduction

The benefits of physical activity (PA) has become a leading topic of study in sports and health sciences since physical inactivity is a leading contributor to global mortality rates [1]. The beneficial outcome of physical activity is widely known. Several researchers have found that regular exercise is one of the effective ways to maintain and improve physical fitness and health; it increases physiological functions and has a positive impact on mental health and the prevention of many chronic diseases [2,3,4]. Additionally, physical activity helps delay the aging process and increases subjective well-being in all ages [5,6]. It also has a beneficial effect on body composition. It is well known that physical inactivity increases the chances of a higher body mass index (BMI) in both genders [7]. For example, a study among US adults confirmed the phenomenon that people with a high level of physical activity had a lower BMI than those who were inactive [8]. In addition to improving individuals’ quality of life, physical activity indirectly affects the performance and competitiveness of the national economy as well [9].

In recognition of this vital link between physical activity and health, the member states of the World Health Organization (WHO) agreed to reduce physical inactivity by 10% by 2025 [1]. The WHO also provides recommendations for levels of physical activity for children, adults, and older adults to achieve significant health benefits and mitigate health risks. According to the recommendations, all adults should undertake at least 150–300 min of moderate-intensity aerobic physical activity or at least 75–150 min of high-intensity aerobic exercise per week for substantial health benefits.

The European Union (EU) has been monitoring countries’ physical activity levels with the Special Eurobarometer 472 since 2009. The latest data showed that nearly half of Europeans (46%) did not engage in any physical activity or sport, and that proportion has increased gradually in recent years. A similar tendency can be found in the Hungarian population as well. Furthermore, 53% did not engage in any physical activity in Hungary, which is a 9% increase on previous data [10]. In addition to the regularity of PA, it is also necessary to investigate the level of physical activity. The short form of the International Physical Activity Questionnaire (IPAQ-SF) has been used to assess individuals’ physical activity levels. It differentiates between vigorous, moderate, and walk levels of PA. Recently, the reliability and validity of the IPAQ-SF were tested. A significant correlation was found between self-reported and accelerometry-based physical activity [11,12].

The 2017 Eurobarometer data showed that 57% of the Hungarian population did not engage in vigorous physical activity, which is close to the EU 28’s average [10]. However, they are a little more likely to engage in vigorous physical activity than EU citizens on average. Moderate physical activity shows even better trends: 43% of the Hungarian population never engage in moderate physical activity, while the EU figure is 47%. At the same time, 4–7 days of moderate-intensity exercise was achieved by more people in Hungary than in the EU (28% compared to 23%). The ratios are reversed for light-intensity PA: 20% of the Hungarian people do not walk at all for at least 10 min at a time, compared to 15% in the EU. Hence, it is interesting to note that more regular participation in vigorous and moderate physical activity seems to characterize the Hungarian population more than EU citizens. However, participation in walking was significantly lower for Hungarians. Thus, it seems the Hungarian population engages less in easier exercise, but people who regularly take part in some form of physical activity do this at a higher intensity and more frequently than the European average. Previous Hungarian studies showed a relatively high level of vigorous physical activity (over 61–63%), but these studies might not present a realistic picture of the population due to their data collection [13,14]. Even though there is some improvement in physical activity, the Hungarian population is still one of the most overweight countries in the European Union. According to Rurik and his colleagues [15], more than two-thirds of the Hungarian population are overweight or obese.

The influence of sociodemographic factors on physical activity was proven in previous studies. According to the Hungarian National Population Health Survey [16], the individuals engaging in regular physical activity were characterized as male, younger adult, higher educated, and single, with a high socioeconomic status [13,17]. Low socioeconomic status reduces the willingness and the opportunities to participate in sports or physical activity, since an essential precondition for physical activity is the availability of free time, disposable income, reasonable living standards, and the access to different sports services [18]. Gerovasili and his colleagues [19] found that men of higher education and higher income are associated with a higher level of physical activity. Ortiz-Hernández and Ramos-Ibáñez [20] state that physical activity decreases with age and women with high socioeconomic status engage in less physical activity than women with low socioeconomic status. Studies are available on the differences between rural-urban physical activity levels are inconsistent. For example, according to Dumith and his colleagues [21], physical activity was more prevalent among wealthier and urban populations. Fan and Cao [22] reported higher levels of moderate to vigorous physical activity (MVPA) in urban areas among Chinese students. However, Brzęk and her colleagues [23] found that people living in the countryside have higher physical activity than those living in towns. Kostka and his colleagues [24] concluded the same when investigating older adults. Attributes of the built environment have been related to PA engagement and obesity levels. Based on the available evidence, fostering suitable urban environments might be critical to sustaining physical activity behaviors [25,26].

In addition to the impact of sociodemographics on physical activity, there is a clear pattern in physical inactivity in different geographic regions within countries [27]. However, the Eurobarometer 472 only focuses on physical activity at the country level. We have little regional level data on physical activity in Hungary and other countries. It is important to note that studies on the Hungarian population show that dynamically developing regions [17] have a positive impact on PA.

Thus, it would be interesting to see the level of physical activity in different geographic regions in Hungary. Therefore, our aim is twofold. First, we would like to examine the physical activity on the Southern Great Plain (NUTS2 level) region of Hungary, including three counties (Csongrád, Békés, and Bács-Kiskun). Second, our goal is to explore the relationship between gender, age, education, income, place of living, and BMI with levels of physical activity.

## 2. Materials and Methods

### 2.1. Sample and Procedure

The data collection occurred at different face-to-face events from July 2018 to January 2019. A total of 83 events were held in this period. It included 14 major events (e.g., “Fit Family” family health sports days and “Fit Bath” health sports programs on the beach) and 69 small events (e.g., “Senior Fitness Days” for seniors and “Exercise Hour” for employees). The participants were able to engage in different types of recreational physical activities and completed our paper-based survey voluntarily. Before data collection, ethical approval was sought and obtained from the university’s Institutional Review Board (Ethical number: 2/2019-SZTE). The survey was voluntary, and it took approximately 5 min to complete. In the survey, the participants were asked about their socioeconomic background and their physical activity. It was ensured that the data were collected anonymously, and that no identifying data, such as names, were collected. After data cleaning, a total of 1648 participants (Men = 572; Women = 1076) were involved in this study. Their mean age was 43.0 (SD = 15.3). All of them were inhabitants in a Southern Great Plains settlement.

### 2.2. Measures

#### 2.2.1. Sociodemographic Questions

Social and demographic data were collected from the participants. The survey included closed-ended questions about age, gender, and educational background (answer categories: Primary school; Vocational school; Secondary school; University). We asked participants about their place of residence. The settlement categories were aligned to official categorization (answer categories: Village; Small- or medium-size town; City). The income per capita in households of the participants was investigated as well (answer categories: below 100,000 HUF; 100,000–200,000 HUF; 200,000–400,000 HUF; above 400,000 HUF). Furthermore, we asked about participants’ height (m) and weight (kg) whereof BMI was calculated using BMI formula (kg/m^2^).

#### 2.2.2. International Physical Activity Questionnaire

Physical activity was measured with the short form of the Hungarian version of the International Physical Activity Questionnaire (IPAQ-SF) [28]. The self-administered questionnaire included seven items, which measured the frequency (day/week) and length (hour/minutes) of walking and moderate- and vigorous-intensity physical activity during the previous seven days (e.g., “How many days did you perform vigorous physical activity that made you feel tired or breathless during the past week?” [29] (p. 2)). The seventh item of the IPAQ-SF questionnaire was an additional indicator to measure sedentary behavior, but this item was not included as part of the summary score of physical activity; hence, we did not use this item in this study.

To create physical activity levels, we followed the official IPAQ scoring protocol (www.ipaq.ki.se). We calculated the metabolic equivalent of task (MET), which is used to estimate the energy expenditure of physical activity. The IPAQ scoring protocol suggests the following equation to calculate a participant’s metabolic equivalent:

- Vigorous MET-minutes/week = 8.0 * minutes of activity per day * days of activity per week;
- Moderate MET-minutes/week = 4.0 * minutes of activity per day * days of activity per week;
- Walking MET-minutes/week = 3.3 * minutes of activity per day * days of activity per week.

After calculating the sample’s MET score, we classified them into low, moderate, and high physical activity levels:

1. High level of physical activity—participants who spend three or more days of vigorous activity accumulating at least 1500 MET min/week or had seven days of any combination of walking, moderate-intensity, or vigorous-intensity activities achieving a minimum of 3000 MET min/week.
2. Moderate intensity—participants who had three or more days of vigorous exercise for at least 20 min/day or five or more days of moderate-intensity activity or walking for at least 30 min/day or five or more days of any combination of walking, moderate-intensity, or vigorous-intensity activities, achieving a minimum of 600 MET min/week.
3. Low level of physical activity—participants who did not have any exercise or did not have enough to meet the moderate and high categories [30] (p. 3).

#### 2.2.3. Data Analysis

SPSS for Mac 25.0 (Statistical Package for Social Sciences) was used for statistical analysis. Descriptive statistics, including frequencies (percentage), means, and standard deviation, were used to assess the sample’s characteristics. Significant gender differences were investigated with the chi-square test (X^2^). Multinominal logistic regression with an odds ratio (OR) and a 95% confidential interval (CI) was used to understand more about the relationship between physical activity and sociodemographic. Several potential determinants were included in this analysis, such as gender, age, residential, education, income, and BMI. In the multinominal regression model, low physical activity was used as a reference category, and differences in moderate physical activity and high physical activity were calculated.

## 3. Results

Table 1 represents the gender-specific characteristics of our sample. Most of the participants represented the 30–40 age groups (43.9%) and came from one of the small towns of the Southern Great Plain region of Hungary (42.2%). Moreover, 36.7% of the participants had a high school degree, but most of the sample had a degree (42.7%). Half of the sample had HUF 100,000–200,000 (EUR = 282–565) income per capita in their households. We calculated each participant’s BMI. Only 45.2% of them had normal BMI, and the average of the sample was 25.90 (SD = 4.62). The level of physical activity showed that 19.2% of the participants had low, 41.1% had moderate, and 39.7% of them had a high level of physical activity. The total MET of the sample was 2063.61 MET min/week (SD = 1692.49 MET min/week). The gender-specific MET values were 2440.41 MET min/week for men and 1864.30 MET min/week for women. The sociodemographic variable showed significant gender differences in age, residential, education, income, BMI, and physical activity level (see Table 1).

In the next step of our analysis, we used multinominal regression to determine the sociodemographic variable between the moderate and the low physical activity groups (Table 2). According to the model, men had higher odds to moderate physical activity than women (OR = 1.56; CI = 1.13–2.15). Younger adults were 1.82 times more likely to participate in moderate physical activity than the age above 65 (OR = 1.82; CI = 1.00–3.29). We found a significant decrement in moderate to low physical activity in the age group of 30 and 49 (OR = 0.77; CI = 0.47–1.26) compared with the older age above 65. Those living in a small town had lower odds (OR = 0.68; CI = 0.47–0.97) to participate in moderate physical activity than those living in cities. Education also had an impact on moderate physical activity. Those who had vocational school qualifications (OR = 0.54; CI = 0.36–0.84) were less likely to have moderate PA than participants with a university degree. A similar result was found in the comparison between high and low physical activity. The men had 2.77 higher odds to participate in high physical activity than women. Younger adults (age 18–29) had 2.51 higher odds compared to the age group of 65+. Residents of small towns had an odds of 0.68 to participate in high physical activity, unlike participants from cities. Those who had vocational school qualifications were less likely to have high levels of physical activity than participants with university degrees (OR = 0.61; CI = 0.40–0.94). Normal BMI was found to be associated with the increased high level of physical activity (OR = 1.48; CI = 1.00–2.20).

In the last step of our analysis, gender-specific multinominal regression was used to see the gender differences against the sociodemographic variables (Table 3). Ages between 18 and 29 was associated with a high level of physical activity among men (OR = 4.40; CI = 1.13–2.15). Women from small towns had lower odds of participating in moderate PA than those from cities (OR = 0.64; CI = 0.41–0.98). Vocational education was found to be associated with moderate physical activity in both genders. Primary school education meant lower odds of high intensity of physical activity among women (OR = 0.42; CI = 0.19–0.90). Normal BMI showed an increased probability of high-intensity physical activity compared to obese women (OR = 1.66; CI = 1.03–2.65).

## 4. Discussion

The present study aimed to investigate the level of physical activity in the Southern Great Plain region of Hungary and explore the relationship between sociodemographics, BMI, and physical activity among the adult population. This study is the first to investigate the Southern Great Plain region using IPAQ with a large sample of populations.

It seems that the population in this region had a relatively lower level of physical activity compared to the results of other Hungarian studies, but this is also due to the different sampling procedure. As reported, the participants showed 39.7% high, 41.1% moderate, and 19.2% low levels of physical activity in our study, while Bácsné [14] reported a 63.40% high level of physical activity in a large Hungarian sample. Makai and her colleagues [13] also found a high value of vigorous PA by surveying the adult population in two counties. Nevertheless, our result is still more favorable than the global trends [31]. Importantly, an early study of Paulik and his colleagues [17] found that participating in regular physical activity was the highest in the Southern Great Plain compared to other Hungarian regions; however, they also used different methods. Thus, it is not easy to draw a conclusion from this result. To improve understanding on physical activity in the Southern Great Plain, it is essential to understand the peculiarities and sociodemographic of this region.

By investigating the relationship between sociodemographics, BMI, and physical activity, we found that gender, age, place of residence, and education were associated with a moderate and high level of PA, while BMI was found to be associated with only the high level of physical activity. Furthermore, our gender-specific analysis revealed that women with normal BMI, living in urban places, holding a university degree, as well as men of a younger age and with higher education, had a higher probability of moderate or high level of physical activity.

Our result was not surprising, and it was consistent with the phenomenon that men had a higher willingness and motivation to participate in any physical activity than women [32]. As it was shown, men had higher odds on both moderate and high levels of physical activities than women. We found that the age group of 18–29 years was the most active. Several studies have shown that physical activity decreases with age and interventions are required in the younger age groups [33]. However, in our study, the age group of 30–49 had significantly lower odds of participating in moderate physical activity than the age group of 65. Interestingly, according to our gender-specific analysis, age was only found as a significant factor for men, but it also transpired that physical activity was lower for the age group of 30 to 49 than above 65 in both genders. Bélanger et al. [34] found similar results surveying British men and women. We believe that physical activity in the lower age groups might have time constraints (e.g., work, family), and older adults might return to their active lifestyle. Only the analysis of high vs. low physical activity of men showed higher levels in that age group, but we believe that this is because more men have manual labor jobs than women in this age group [35].

Living in small towns and villages was found to result in lower odds to participate in a moderate and high level of physical activity, which is inconsistent with previous studies on the Hungarian population [14,17]. These findings reinforce research findings that emphasize the correlation between urbanization and higher levels of physical activity [36]. Our gender-specific analysis showed the same result. Even though results pertaining to men were less significant than those pertaining to the women, both genders showed lower odds to participate in physical activity in small settlements. We assume that these people might commute to their work daily, presumably not in an active way, and they might have less time for regular exercise.

Several studies have shown the associations between physical activity and education [19]. It is widely known that higher educational background indicates a higher level of physical activity, among other positive factors. In our results, the participants with the vocational school qualifications had lower odds to participate in a moderate and high level of PA than participants with a university degree. These results were reinforced in the gender-specific analysis as well. Gerber et al. [37], by surveying Swiss vocational students, found that nearly half of the students did not meet the MVPA recommendations. It appears that vocational schools spend less time promoting regular physical activity, and our results also reflect this phenomenon.

The relationship between body composition and physical activity is well established in the literature for all ages [38]. Not surprisingly, we found that individuals with a normal BMI are more likely to engage in a high level of physical activity. As it was mentioned earlier, two-thirds of the Hungarian population are overweight or obese, which negatively influences physical activity [15]. Although our results showed some improvement from previous studies, more than half of the participants were still considered overweight or obese. Our gender-specific analysis highlighted that women with a normal BMI had 1.66 higher odds of participating in a high level of physical activity than obese women. The reason behind this result might be the gender differences related to BMI. Previous studies and our results showed that men have a higher BMI than women [15]. Thus, we believe that the positive effects of BMI on physical activity levels are more pronounced for women.

Income was not associated with physical activity, although a previous study on this topic found income to be a significant predictor variable [19]; those with higher incomes are more willing to spend on sports [39,40]. Thus, variables, such as education and place of residence, played a more prominent role in this study, although further analysis is needed for the future.

Our study presents a few limitations that need to be addressed here. First, the data were collected at designated events that participants attended voluntarily; thus, our results may not be generalized. Second, the distribution of the different variables was not even. For example, only 572 men participated compared to 1076 women. A similar gap can be found in age, place of residence, education, and income as well. To remove these limitations, our study will continue in the future. Our goal is to increase our sample and involve more people in the Southern Great Plain region of Hungary.

## 5. Conclusions

We described the characteristics of physical activity in the Southern Great Plain region of Hungary among the adult population. We analyzed BMI and sociodemographic predictors, such as age, gender, education, residence, and income, and we concluded the following: (1) 39.7% of the respondents belonged to the category of high physical activity and 19.2% to the category of low physical activity; (2) younger men had higher odds of engaging in a high level of physical activity than women in any age group; (3) people living in rural places had lower odds of participating in moderate or high physical activity levels; (4) vocational school qualifications lowered the chance of a high and moderate level of physical activity; and (5) normal BMI was associated with a high level of physical activity.

To sum up, we believe that our study provided valuable data on physical activity in Hungary. This study might create the opportunity to continue our project and expand our sample size to improve understanding around what drives people to engage in physical activity in this region. Additionally, this information might help experts to maintain or increase participation in physical activity.

## Figures and Tables

**Table 1:** Sociodemographics, BMI, and physical activity of the participants.

	Total (*n* = 1648)	Men (*n* = 576)	Women (*n* = 1076)	X^2^
Age				10.16 *
18–29	23.3% (*n* = 384)	27.1% (*n* = 155)	21.3% (*n* = 229)	
30–49	43.9% (*n* = 724)	40.2% (*n* = 230)	45.9% (*n* = 494)	
50–65	23.5% (*n* = 387)	22.2% (*n* = 127)	24.2% (*n* = 260)	
65+	9.3% (*n* = 153)	10.5% (*n* = 60)	8.6% (*n* = 93)	
Place of Residence				8.20 *
Village	32.9% (*n* = 542)	34.4% (*n* = 197)	32.1% (*n* = 345)	
Small town	42.2% (*n* = 712)	38.6% (*n* = 221)	45.6% (*n* = 491)	
City	23.9% (*n* = 394)	26.9% (*n* = 154)	22.3% (*n* = 240)	
Education				24.77 ***
Primary School	6.7% (*n* = 100)	5.6% (*n* = 32)	7.2% (*n* = 78)	
Vocational school	13.8% (*n* = 227)	19.4% (*n* = 111)	10.8% (*n* = 116)	
High school	36.7% (*n* = 604)	33.2% (*n* = 190)	38.5% (*n* = 414)	
University	42.9% (*n* = 707)	41.8% (*n* = 239)	43.5% (*n* = 468)	
Household Income per Head (HUF)				31.18 ***
100,000	19.1% (*n* = 314)	16.4% (*n* = 94)	20.4% (*n* = 220)	
100,000–200,000	50.2% (*n* = 827)	47.6% (*n* = 272)	51.6% (*n* = 555)	
200,000–400,000	24.2% (*n* = 399)	25.0% (*n* = 143)	23.8% (*n* = 256)	
400,000	6.6% (*n* = 108)	11.0% (*n* = 63)	4.2% (*n* = 45)	
BMI				38.18 ***
Underweight	1.9% (*n* = 31)	0.7% (*n* = 4)	2.5% (*n* = 27)	
Normal	45.2% (*n* = 745)	37.1% (*n* = 212)	49.5% (*n* = 533)	
Overweight	34.2% (*n* = 563)	42.7% (*n* = 244)	29.6% (*n* = 319)	
Obese	18.8% (*n* = 309)	19.6% (*n* = 112)	18.4% (*n* = 197)	
Physical Activity Level				48.40 ***
Low	19.2% (*n* = 317)	12.9% (*n* = 74)	22.6% (*n* = 243)	
Moderate	41.1% (*n* = 677)	36.5% (*n* = 209)	43.5% (*n* = 468)	
High	39.7% (*n* = 654)	50.5% (*n* = 289)	33.9% (*n* = 365)	

Notes. *** < 0.001; * < 0.05.

**Table 2:** Sociodemographic factors associated between low, moderate, and high physical activity.

	Moderate vs. Low		High vs. Low	
	OR	95% CI	OR	95% CI
Gender				
Men	1.56 *	1.13–2.15	2.77 ***	2.01–3.82
Women	1.00		1.00	
Age				
18–29	1.82 *	1.00–3.29	2.51 *	1.36–4.62
30–49	0.77 *	0.47–1.26	0.84	0.50–1.42
50–65	0.94	0.56–1.58	0.94	0.54–1.63
65+	1.00		1.00	
Place of Residence				
Village	0.73	0.49–1.07	0.68	0.45–1.00
Small town	0.68 *	0.47–0.97	0.68 *	0.47–1.40
City	1.00		1.00	
Education				
Primary School	0.95	0.54–1.67	0.57	0.30–1.08
Vocational school	0.54 *	0.36–0.84	0.61 *	0.40–0.94
Secondary school	0.87	0.63–1.21	1.01	0.73–1.40
University	1.00		1.00	
Household Income per Head (HUF)				
100,000	1.34	0.70–2.59	0.89	0.46–1.72
100,000–200,000	1.35	0.74–2.47	1.26	0.70–2.29
200,000–400,000	1.13	0.60–2.11	1.03	0.55–1.91
400,000	1.00		1.00	
BMI				
Underweight	1.13	0.41–4.62	0.56	0.18–1.79
Normal	1.22	0.83–1.79	1.48 *	1.00–2.20
Overweight	1.02	0.70–1.48	0.92	0.62–1.35
Obese	1.00		1.00	

Notes. *** < 0.001; * < 0.05.

**Table 3:** Gender specific associations between low, moderate, and high physical activity.

	Men				Women			
	Moderate vs. Low		High vs. Low		Moderate vs. Low		High vs. Low	
	OR	95% CI	OR	95% CI	OR	95% CI	OR	95% CI
Age								
18–29	2.46	0.84–7.20	4.40 *	1.49–12.97	1.63	0.79–3.36	1.68	0.79–3.53
30–49	0.92	0.39–2.17	1.59	0.67–3.82	0.75	0.41–1.39	0.53	0.27–1.01
50–65	1.03	0.41–2.60	1.62	0.63–4.15	0.92	0.49–1.75	0.60	0.30–1.19
65+	1.00		1.00		1.00		1.00	
Place of Residence								
Village	0.91	0.43–1.89	0.58	0.28–1.19	0.64	0.40–1.01	0.77	0.47–1.25
Small town	0.76	0.37–1.57	0.77	0.38–1.55	0.64 *	0.41–0.98	0.66	0.42–1.04
City	1.00		1.00		1.00		1.00	
Education								
Primary School	1.15	0.32–4.10	0.91	0.25–3.34	0.90	0.47–1.72	0.42 *	0.19–0.90
Vocational school	0.50 *	0.23–1.06	0.59	0.29–1.22	0.56 *	0.33–0.95	0.66	0.37–1.13
Secondary school	1.09	0.52–2.27	1.53	0.75–3.12	0.86	0.59–1.24	0.88	0.59–1.28
University	1.00		1.00		1.00		1.00	
Household Income per Head								
100,000	2.15	0.71–6.53	1.49	0.49–4.49	1.13	0.45–2.79	0.61	0.24–1.49
100,000–200,000	1.59	0.64–3.96	1.89	0.78–4.62	1.22	0.51–2.88	0.82	0.35–1.91
200,000–400,000	1.02	0.40–2.61	1.07	0.43–2.67	1.11	0.45–2.71	0.79	0.32–1.91
400,000	1.00		1.00		1.00		1.00	
BMI								
Underweight	0.74	0.57–9.60	0.21	0.01–4.14	1.18	0.38–3.60	0.70	0.19–2.48
Normal	1.16	0.54–2.50	1.25	0.59–2.62	1.20	0.77–1.86	1.66 *	1.03–2.65
Overweight	1.36	0.67–2.76	1.25	0.63–2.47	0.92	0.59–1.42	0.83	0.50–1.35
Obese	1.00		1.00		1.00		1.00	

Note. * < 0.05.

## Data Availability

The data presented in this study are available on request to qualified researchers from the corresponding author.
